# Supplementary material for: Structures of Coxsackievirus A10 unveil the molecular mechanisms of receptor binding and viral uncoating
Source: Nat Commun. 2018 Nov 26;9:4985. doi: 10.1038/s41467-018-07531-0 (PMC6255764; doi:10.1038/s41467-018-07531-0)
Supplement: Supplementary file 1 — Supplementary Information [file 41467_2018_7531_MOESM1_ESM.pdf]

Supplementary Information

Structures of Coxsackievirus A10 unveil the molecular mechanisms of receptor binding and viral uncoating

L. Zhu, Y. Sun, J. Fan et al.

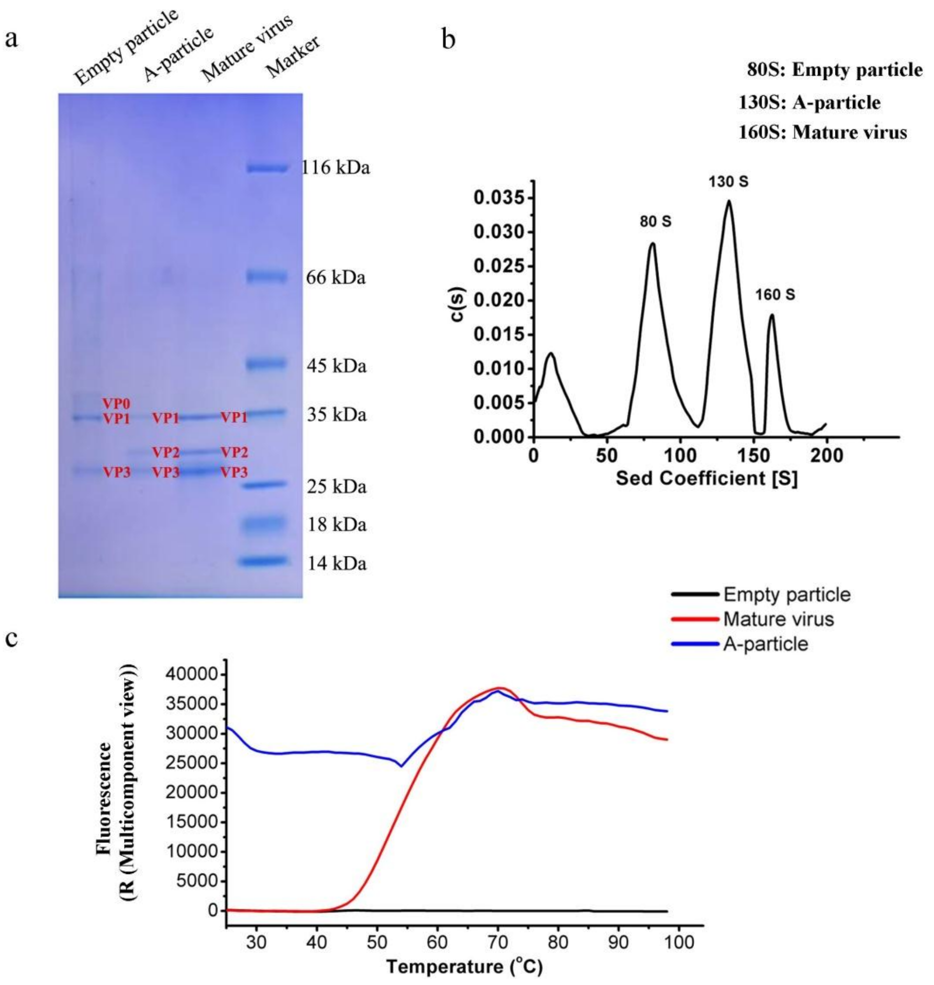

Supplementary Figure 1

Characterization of the three types of CVA10 particles.

(a) Protein compositional analysis of the three types of CAV10 particles by NuPAGE 4–12% Bis-Tris Gel (Invitrogen). **(b)** Analytical ultracentrifugation. The experiments were carried out at 20 ℃, which produced three major peaks with sedimentation coefficients of 80S, 130S and 160S and these are referred to as empty particle, A-particle and mature virus, respectively. **(c)** PaSTRy assay. To detect RNA exposure and characterize the stability of the three types of CAV10 particles, a dye used to detect RNA, named SYTO9, was applied to perform differential scanning fluorimetry assays. The raw fluorescence traces of CAV10 mature virus, A- and empty particles incubated with SYTO9 are shown, indicating that the empty particles contain no detectable RNA, while mature viruses and A-particles do contain RNA.

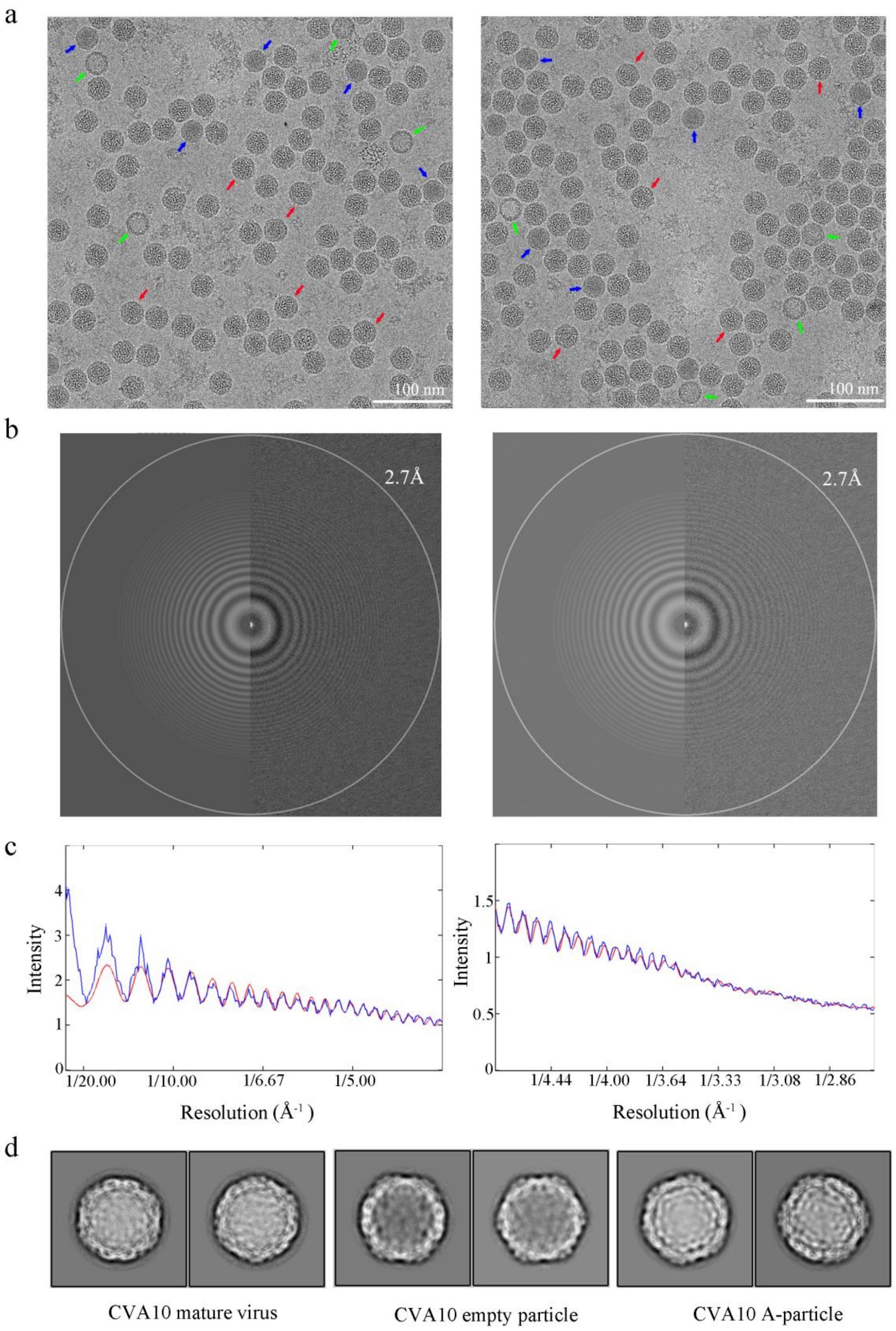

Supplementary Figure 2

Cryo-EM images and 2D classification of the three types of CVA10 particles.

(a) Selected cryo-EM images. Blue, green and red arrows mark CVA10 mature virus, empty- and A-particles, respectively. **(b)** The corresponding Fourier transforms. **(c)** Fit of the CTF estimate from one selected image. Left: low spatial frequencies from 20 Å to 3.8 Å; right: high spatial frequencies from 4.8 Å to 2.7 Å. The blue and red curves present circularly averaged power spectrum and simulated CTF, respectively. **(d)** Representative classes from 2D classification in Relion[1].

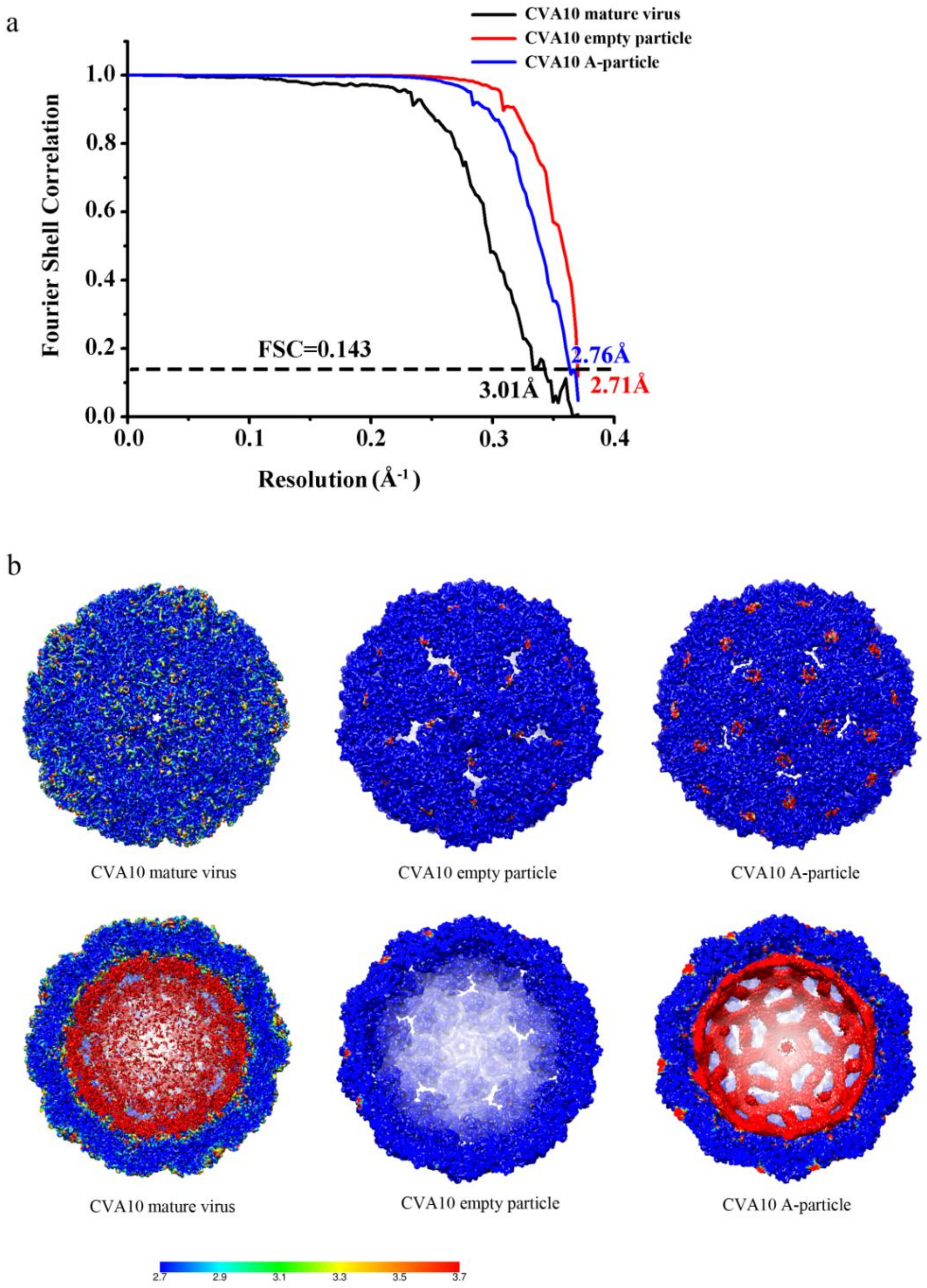

Supplementary Figure 3

Resolution evaluations for EM maps of the three types of CVA10 particles.

(a) The gold standard FSC curves of the final maps of CVA10 mature virus, empty particle and A-particle with a resolution of 3.01 Å, 2.71 Å, and 2.76 Å at FSC=0.143, respectively. **(b)** The final maps were analyzed by ResMap[2], showing resolution distribution from 2.7 to 3.7 Å. Most of the capsids have resolutions better than 2.8 Å, and the resolutions of the interior parts of the map (corresponding to the RNA genome) are ~8–30 Å.

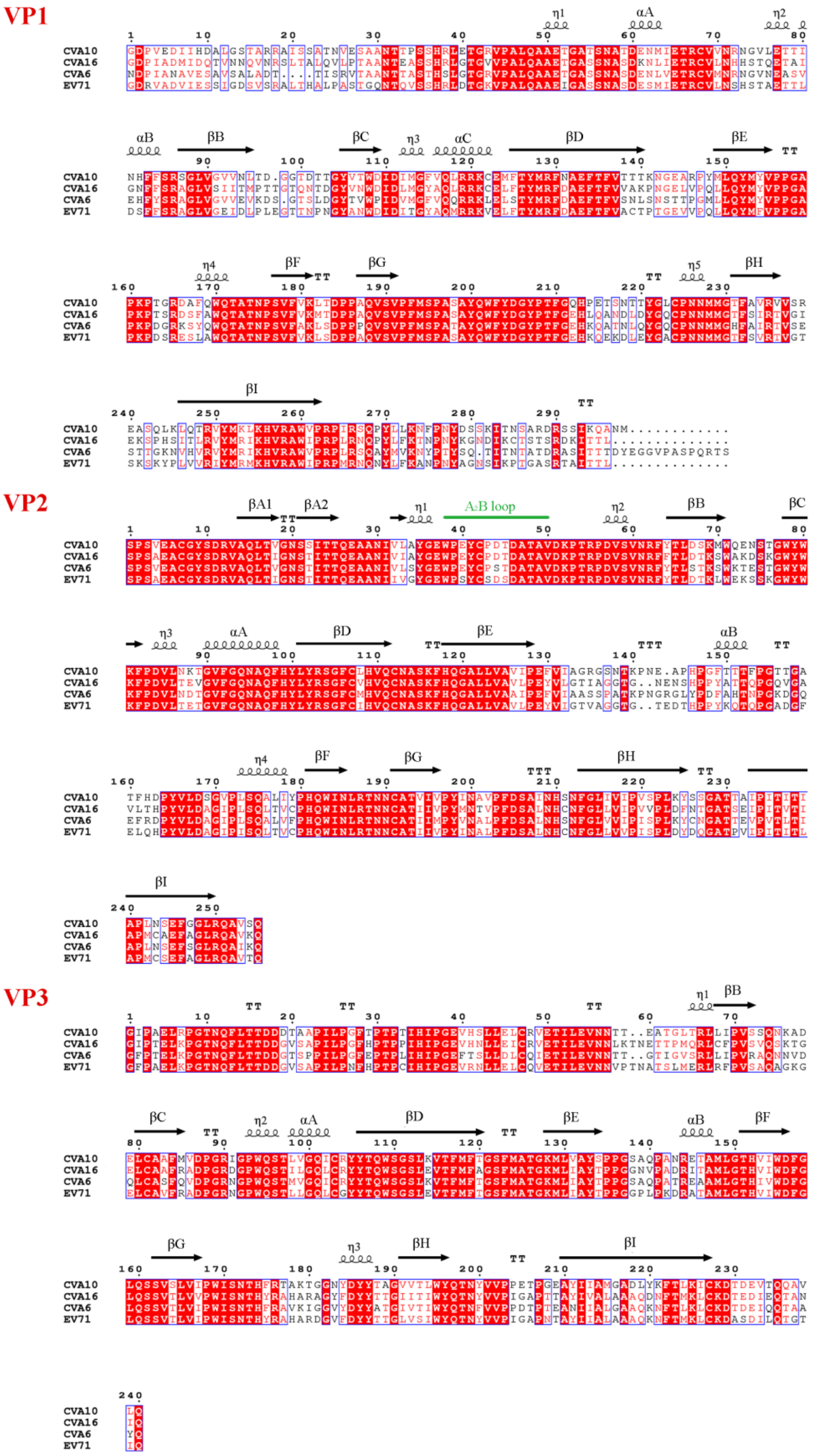

Supplementary Figure 4

Espript[3] representation of sequence alignments of VP1, VP2 and VP3 of CVA10 with CVA6, CVA16 and EV71. Secondary structural elements are labeled.

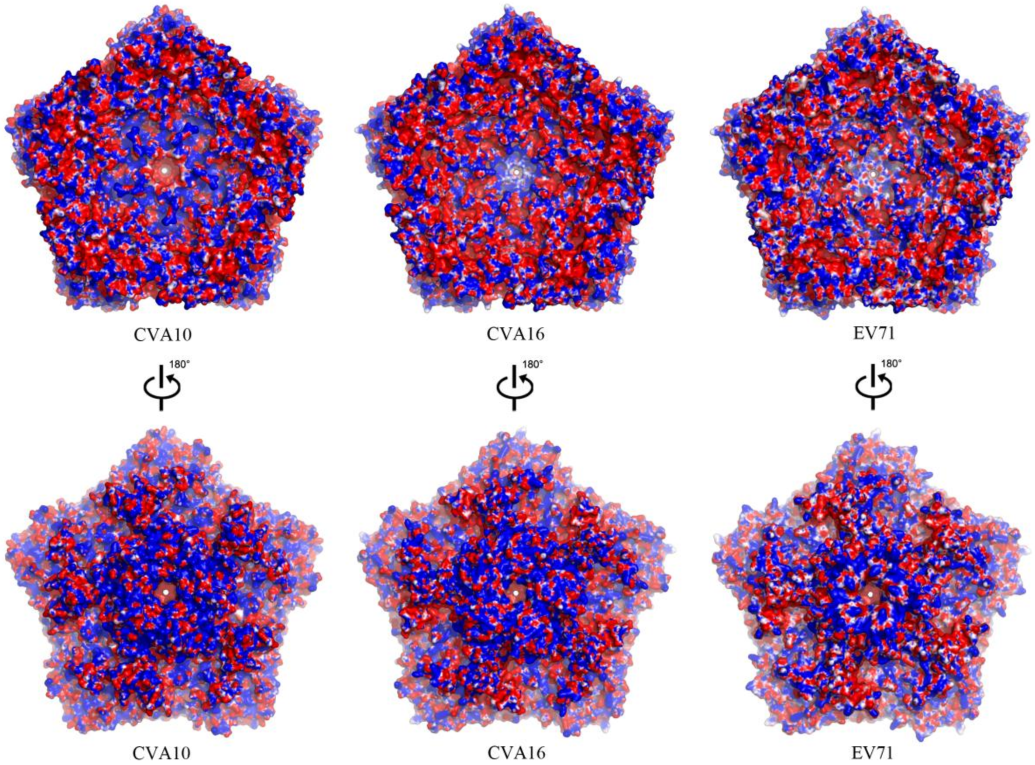

Supplementary Figure 5

Electrostatic surfaces of CVA10, CVA16 and EV71 mature viruses displayed on pentamers of the respective structures. These were calculated using PYMOL[4]; red represents a negative charge and blue a positive charge. Bottom views for the upper panel, top views the bottom panel.

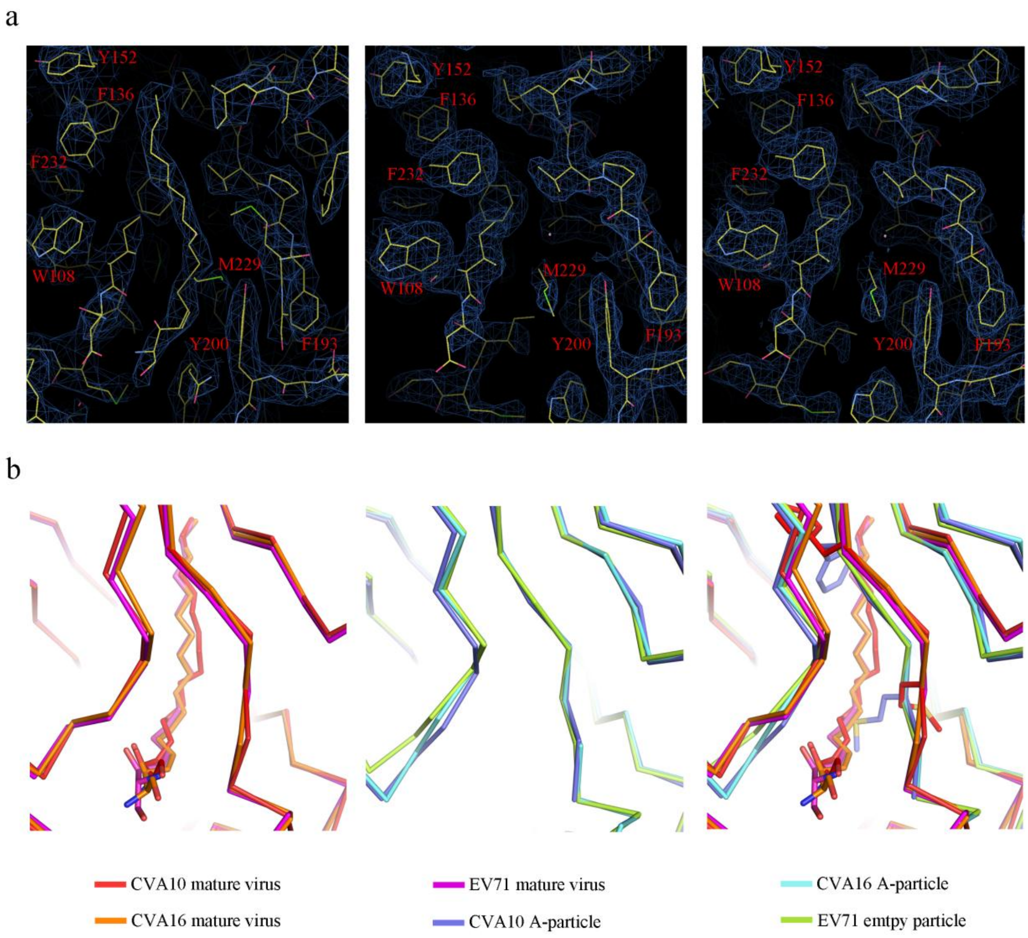

Supplementary Figure 6

Electron density maps and close-up view of the pockets.

(a) Electron density maps, showing electron densities in the pocket factor binding region of VP1 in the mature virus (left), empty- (middle) and A-particles (right). **(b)** Close-up view of the pockets. Left: mature viruses of CVA10 (red), EV71 (magenta) and CVA16 (orange); middle: CVA10 A-particle (light blue), EV71 expanded empty particle (light green) and CVA16 A-particle (cyan); right: superimpositions of these six particles. The βC and βH strands are presented in ribbon format. Bulky side chains in CVA10 A-particle shown as sticks occlude the pocket.

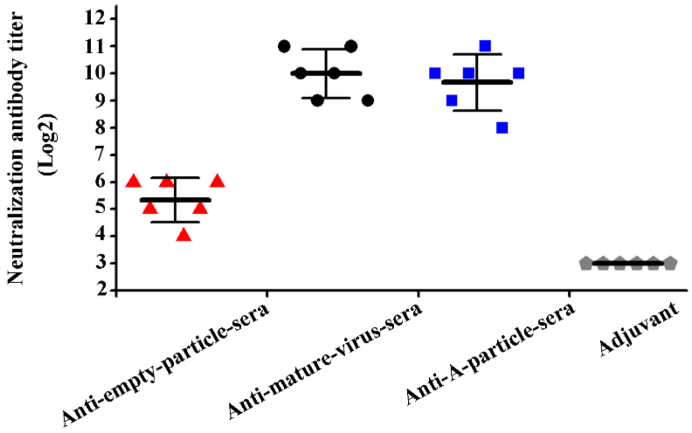

Supplementary Figure 7

***In vitro* neutralizing titer of antisera of the three types of CVA10 particles.** The levels of neutralization antibody titer of all mice from four groups (n = 6 per group, 3 male and 3 female) were plotted (mean ± s.d.).

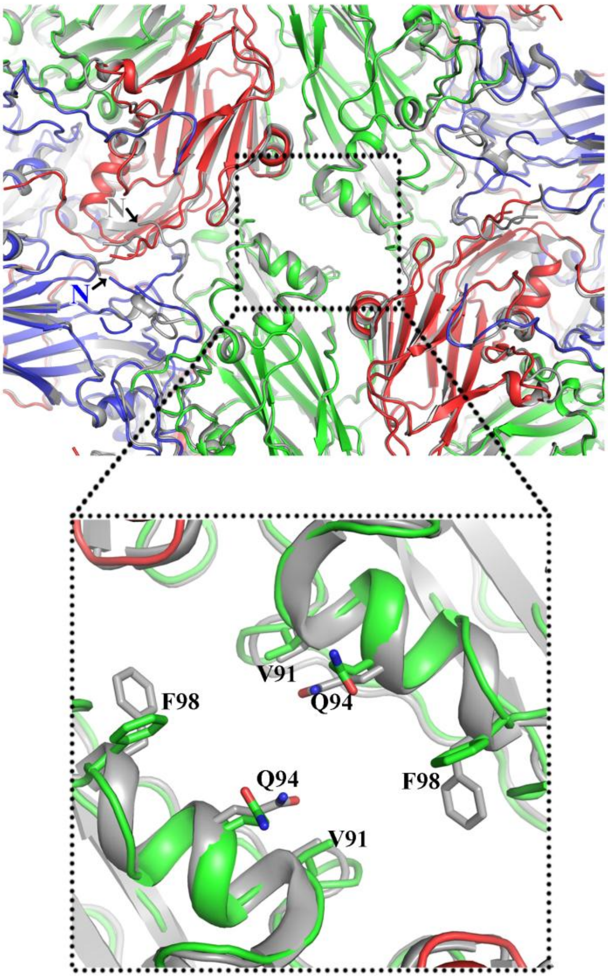

Supplementary Figure 8

Superimposition of the atomic models from CVA10 and CVA16 mature viruses centered at the two-fold axes. The major two-fold channels are marked by dotted rectangles. The color scheme for CVA10 is same as in Fig. 3c, while CVA16 is represented in gray. The VP1 N-terminuses of CVA10 and CVA16 are marked as arrows and labeled. Inset represents the zoomed-in view of the major two-fold channels and Key residues are shown as sticks.

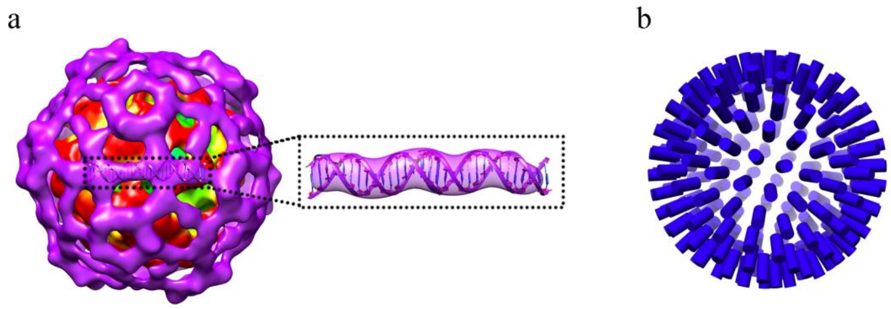

Supplementary Figure 9

Genome densities and angular distribution analysis for the final asymmetrical reconstruction.

(a) Genome densities and zoomed-in view of a dsRNA fragment. **(b)** Angular distribution for the final asymmetrical reconstruction. Each column represents one view and the size of the column is proportional to the number of particles in that view.

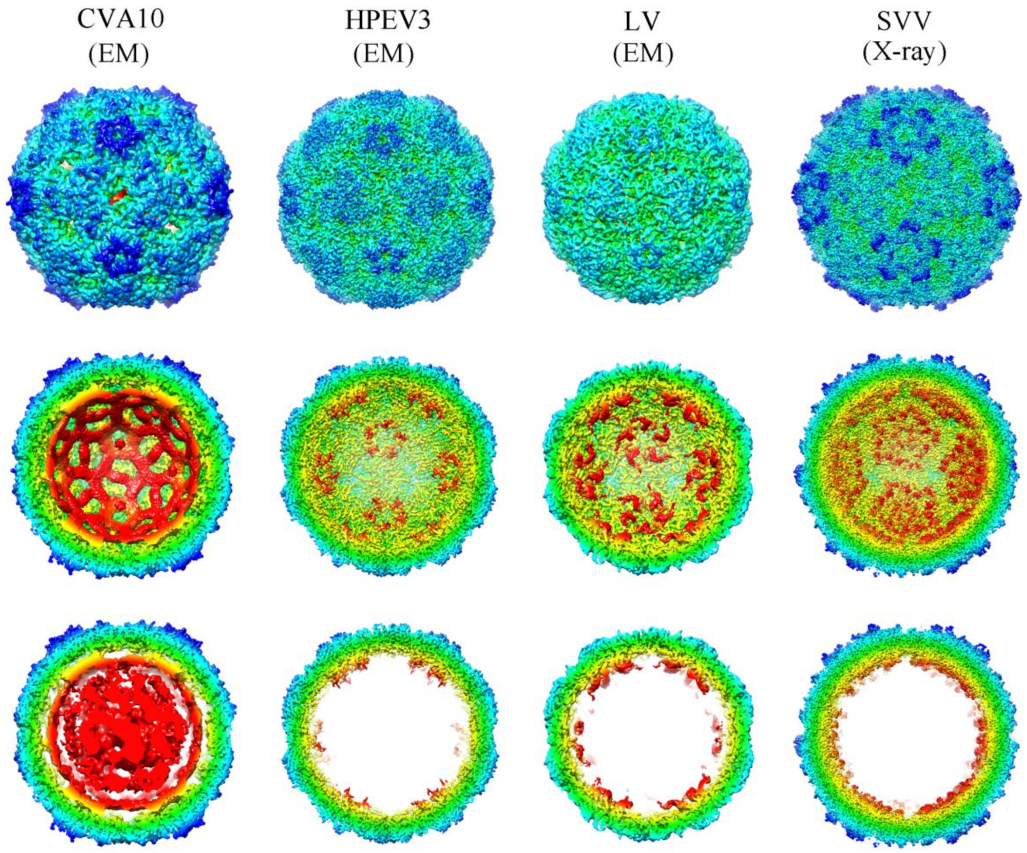

Supplementary Figure 10

Ordered RNA segments in the structures of picornaviruses.

Four representative icosahedrally-symmetric structures for picornaviruses are shown. These are CVA10 A-particle (asymmetric reconstruction), human parechovirus type 3 (cryo-EM, EMD-3137)[5], Ljungan virus (cryo-EM, EMD-6395)[6] and Seneca Valley virus (X-ray crystallography, PDB: 3CJI)[7]. Top row: an exterior view of each capsid, viewed perpendicular to an icosahedral two-fold axis; middle row: the rear half of each capsid with the same orientation (Note: In order to show the connections between the inner capsid and the outer layer of CVA10 genome clearly, an inner mask with a diameter of ~200 Å was applied); bottom row: a ~60 Å thick central section of each capsid with the same orientation.

Supplementary Table 1

Cryo-EM imaging, data processing and refinement statistics.

Name	Mature virus	Empty particle	A-particle
**Data collection**			
Micrographs (total)	1,200	1,200	1,200
Micrographs (used)	800	800	800
Particles selected	4,835	25,683	22,568
Particles included in final reconstruction	4,586	22,725	21,456
Sampling, Å per pixel	1.35	1.35	1.35
Defocus range (μm)	0.8-2.3	0.8-2.3	0.8-2.3
Resolution (Å) (FSC = 0.143 criterion)	3.0	2.7	2.8
**Model Refinement**			
Clashscore	10.4	10.9	11.2
Rotamer outliers (%)	0.28	0.32	0.22
C-beta deviations	0	0	0
Ramachandran statistics (%)			
Most favored	92.8	92.5	93.0
Allowed	6.8	7.2	6.7
Outliers	0.4	0.3	0.3
R.m.s.deviations			
Bond lengths (Å)	0.009	0.010	0.011
Bond angles (°)	1.066	1.122	1.068
R_{work}/R_{free} (%)	25.6/25.9	24.8/25.1	25.1/25.3

Supplementary References

1. Scheres, S.H. RELION: implementation of a Bayesian approach to cryo-EM structure determination. *Journal of structural biology* 180, 519-530 (2012).
2. Kucukelbir, A., Sigworth, F.J. & Tagare, H.D. Quantifying the local resolution of cryo-EM density maps. *Nature methods* 11, 63-65 (2014).
3. Gouet, P., Courcelle, E. & Stuart, D.I. ESPript: analysis of multiple sequence alignments in PostScript. *Bioinformatics* 15, 305-308 (1999).
4. DeLano, W.L. The PyMOL molecular graphics system. (2002).
5. Shakeel, S. et al. Genomic RNA folding mediates assembly of human parechovirus. *Nat Commun* 8, 5 (2017).
6. Zhu, L. et al. Structure of Ljungan virus provides insight into genome packaging of this picornavirus. *Nat Commun* 6, 8316 (2015).
7. Venkataraman, S. et al. Structure of Seneca Valley Virus-001: an oncolytic picornavirus representing a new genus. *Structure* 16, 1555-61 (2008).
