# Supplementary material for: Structures of Coxsackievirus A10 unveil the molecular mechanisms of receptor binding and viral uncoating
Source: Nat Commun. 2018 Nov 26;9:4985. doi: 10.1038/s41467-018-07531-0 (PMC6255764; doi:10.1038/s41467-018-07531-0)
Supplement: Supplementary file 3 — Reporting Summary [file 41467_2018_7531_MOESM3_ESM.pdf]

nature research

Corresponding author(s): Xiangxi Wang

Reporting Summary

Nature Research wishes to improve the reproducibility of the work that we publish. This form provides structure for consistency and transparency in reporting. For further information on Nature Research policies, see Authors & Referees and the Editorial Policy Checklist.

Statistical parameters

When statistical analyses are reported, confirm that the following items are present in the relevant location (e.g. figure legend, table legend, main text, or Methods section).

n/a	Confirmed	
☐	☒	The exact sample size (n) for each experimental group/condition, given as a discrete number and unit of measurement
☐	☒	An indication of whether measurements were taken from distinct samples or whether the same sample was measured repeatedly
☒	☐	The statistical test(s) used AND whether they are one- or two-sided<br>*Only common tests should be described solely by name; describe more complex techniques in the Methods section.*
☒	☐	A description of all covariates tested
☒	☐	A description of any assumptions or corrections, such as tests of normality and adjustment for multiple comparisons
☒	☐	A full description of the statistics including central tendency (e.g. means) or other basic estimates (e.g. regression coefficient) AND variation (e.g. standard deviation) or associated estimates of uncertainty (e.g. confidence intervals)
☒	☐	For null hypothesis testing, the test statistic (e.g. F, t, r) with confidence intervals, effect sizes, degrees of freedom and P value noted<br>*Give P values as exact values whenever suitable.*
☒	☐	For Bayesian analysis, information on the choice of priors and Markov chain Monte Carlo settings
☒	☐	For hierarchical and complex designs, identification of the appropriate level for tests and full reporting of outcomes
☒	☐	Estimates of effect sizes (e.g. Cohen's d, Pearson's r), indicating how they were calculated
☐	☒	Clearly defined error bars<br>*State explicitly what error bars represent (e.g. SD, SE, CI)*

Our web collection on statistics for biologists may be useful.

Software and code

Policy information about availability of computer code

Data collection	SerialEM
Data analysis	GraphPad Software, SEDFIT software, COOT, Phenix, MOTIONCORR, EMAN package, Gctf, Relion 1.4, CHIMERA, Pymol

For manuscripts utilizing custom algorithms or software that are central to the research but not yet described in published literature, software must be made available to editors/reviewers upon request. We strongly encourage code deposition in a community repository (e.g. GitHub). See the Nature Research guidelines for submitting code & software for further information.

Data

Policy information about availability of data

All manuscripts must include a data availability statement. This statement should provide the following information, where applicable:

- Accession codes, unique identifiers, or web links for publicly available datasets
- A list of figures that have associated raw data
- A description of any restrictions on data availability

The atomic coordinates of CVA10 mature virus, empty- and A-particles have been submitted to Protein Data Bank with accession numbers PDB: 6AKS, 6AKU and 6AKT respectively. Cryo-EM density maps of CVA10 mature virus, empty particle, A-particle and viral genome have been deposited with the Electron Microscopy Data Bank: EMD-9642, EMD-9644, EMD-9643 and EMD-9652 respectively. Other data are available from the corresponding authors upon reasonable request.

Field-specific reporting

Please select the best fit for your research. If you are not sure, read the appropriate sections before making your selection.

☒ Life sciences ☐ Behavioural & social sciences ☐ Ecological, evolutionary & environmental sciences

For a reference copy of the document with all sections, see nature.com/authors/policies/ReportingSummary-flat.pdf

Life sciences study design

All studies must disclose on these points even when the disclosure is negative.

Sample size	Trial experiments done previously were used to determine sample size with adequate statistical power.
Data exclusions	No data were excluded from the analyses.
Replication	For each experiment, three attempts at replication were successful.
Randomization	Gender- and age-matched mice were randomly assigned to groups for in vivo experiments.
Blinding	The investigators were not completely blinded during the experiment.

Reporting for specific materials, systems and methods

Materials & experimental systems

n/a	Involved in the study	
☒	☐	Unique biological materials
☒	☐	Antibodies
☐	☒	Eukaryotic cell lines
☒	☐	Palaeontology
☐	☒	Animals and other organisms
☒	☐	Human research participants

Methods

n/a	Involved in the study	
☒	☐	ChIP-seq
☒	☐	Flow cytometry
☒	☐	MRI-based neuroimaging

Eukaryotic cell lines

Policy information about cell lines

Cell line source(s)	National Institutes for Food and Drug Control
Authentication	Yes, including Morphology, DNA Profile, Mycoplasma test, Cell growth curve and population doubling time.
Mycoplasma contamination	Yes. No mycoplasma contamination.
Commonly misidentified lines (See ICLAC register)	Not Applicable

Animals and other organisms

Policy information about studies involving animals; ARRIVE guidelines recommended for reporting animal research

Laboratory animals	Four-week-old SPF BALB/c mice were used (half male; half female)
Wild animals	Not Applicable
Field-collected samples	Not Applicable
